# (2-Pyrid­yl)[5-(2-pyridyl­carbon­yl)-2-pyrid­yl]methanone

**DOI:** 10.1107/S1600536810033957

**Published:** 2010-09-25

**Authors:** Hong Qiang, Fan Zhang, Zi-jia Wang

**Affiliations:** a Department of Chemistry, Capital Normal University, Beijing 100048, People’s Republic of China

## Abstract

In the centrosymmetric title compound, C_17_H_11_N_3_O_2_, the dihedral angle between the central and pendant pyridyl rings is 50.29 (9)°. In the crystal, mol­ecules stack along the *a* axis by π–π inter­actions between the pyridine rings with centroid–centroid distances of 3.845 (2) Å. The N atom and one of the C atoms of the central ring are disordered by symmetry.

## Related literature

For studies on other pyridinyl-based methanone species, see: Papaefstathiou & Perlepes (2002 ▶); Dendrinou-Samara *et al.* (2003 ▶); Crowder *et al.* (2004 ▶); Chen *et al.* (2005 ▶); Wan *et al.* (2008 ▶).
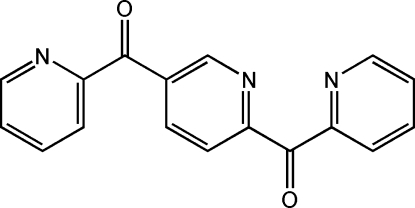


## Experimental

### 

#### Crystal data

C_17_H_11_N_3_O_2_
*M* *_r_* = 289.30
Triclinic, 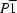

*a* = 3.8453 (13) Å
*b* = 8.447 (3) Å
*c* = 11.202 (3) Å
α = 108.672 (6)°
β = 97.251 (6)°
γ = 99.772 (6)°
*V* = 333.29 (19) Å^3^
*Z* = 1
Mo *K*α radiation
μ = 0.10 mm^−1^
*T* = 293 K
0.60 × 0.50 × 0.29 mm

#### Data collection

Bruker APEXII CCD area-detector diffractometer
Absorption correction: multi-scan (*SADABS*; Bruker, 2007 ▶) *T*_min_ = 0.622, *T*_max_ = 1.000
2301 measured reflections
1623 independent reflections
1198 reflections with *I* > 2σ(*I*)
*R*_int_ = 0.016

#### Refinement

*R*[*F* ^2^ > 2σ(*F* ^2^)] = 0.058
*wR*(*F* ^2^) = 0.177
*S* = 1.07
1623 reflections
100 parameters
H-atom parameters constrained
Δρ_max_ = 0.31 e Å^−3^
Δρ_min_ = −0.26 e Å^−3^

### 

Data collection: *APEX2* (Bruker, 2007 ▶); cell refinement: *APEX2* and *SAINT* (Bruker, 2007 ▶); data reduction: *SAINT*; program(s) used to solve structure: *SHELXS97* (Sheldrick, 2008 ▶); program(s) used to refine structure: *SHELXL97* (Sheldrick, 2008 ▶); molecular graphics: *SHELXTL* (Sheldrick, 2008 ▶); software used to prepare material for publication: *SHELXTL* and *PLATON* (Spek, 2009 ▶).

## Supplementary Material

Crystal structure: contains datablocks I, global. DOI: 10.1107/S1600536810033957/jj2048sup1.cif

Structure factors: contains datablocks I. DOI: 10.1107/S1600536810033957/jj2048Isup2.hkl

Additional supplementary materials:  crystallographic information; 3D view; checkCIF report

## Figures and Tables

**Fig. 1.**
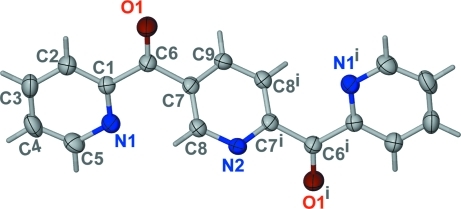
The atom-numbering scheme of the title compound C17H11N3O2. Displacement ellipsoids are drawn at the 50% probability level and H atoms are shown as sticks of arbitrary radii. Symmetry code: i -x + 2, -y + 2, -z.

**Fig. 2.**
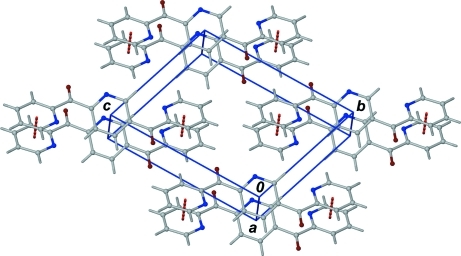
The packing illustration of the title compound, C17H11N3O2. The red-dashed lines indicate weak π···π stacking interactions.

## supplementary crystallographic information

### Comment

Di-2-pyridylmethanone has attracted great interest in recent years as it can exist in various forms in stabilizing its metal complexes, including its neat ketone form, singly and doubly deprotonated gem-diol forms, as well as the monoanion of its hemiacetal form (Papaefstathiou *et al.*, 2002; Dendrinou-Samara *et al.*, 2003; Crowder *et al.*, 2004).  Therefore, homolog compounds such as 2,6-pyridinediylbis(2-pyridyl)methanone (Chen *et al.*, 2005) and 2,6-pyridinediylbis(3-pyridyl)methanone (Wan *et al.*, 2008) were also synthesized and characterized.

In the present study, a new member of this family, namely 2,5-pyridinediylbis(2-pyridyl)methanone (C_17_H_11_N_3_O_2_), is reported. X-ray diffraction analysis shows that the N2 and C9 atoms of the 2,5-pyridinediyl ring have an equal occupancy at the same site.  Thus the molecule is centrosymmetric with two 2-pyridyl methanone groups bonding to the 2,5-pyridinediyl ring at the 2 and 5 positions, respectively.  The 2-pyridyl and the center 2,5-pyridinediyl rings exhibit a dihedral angle of 50.29 (9)° (Fig. 1). Along the *a* axis, the packing between the molecules is provided by weak un-covalent interaction only: /p-electron···/p-electron ring interaction.  The distance between the centroids of the proximate pyridyl rings equals 3.845 (2) Å, as shown in Fig. 2.

### Experimental

The preparation of the title compound followed the procedure previously developed for 2,6-pyridinediylbis(3-pyridyl)methanone (Wan et al., 2008).The crude product was extracted with chloroform, and the combined organic extract was dried over anhydrous sodium sulfate and finally concentrated in vacuo to give a brown oil. Further purification by chromatography on silica gel (R_f_= 0.44, eluent: ether acetate/dichloromethane = 1:6, v/v), giving 2.96 g of light yellow powder of 2,5-pyridinediylbis(2-pyridyl)methanone in 41% yield; m.p. 108-110°C; The yellow crystals of the title compound having a average 0.40 × 0.30 × 0.20 mm dimension were obtained by slow evaporation from its solution of dichloromethane/N,N-dimethylformamide 1/1 (v/v).

### Refinement

The hydrogen atoms were placed in idealized positions and allowed to ride on the relevant carbon atoms, with C—H = 0.93 Å and *U*ĩso~(H) = 1.2*U*~eq~(C).

### Crystal data

**Table:**

C_17_H_11_N_3_O_2_	*Z* = 1
*M**_r_* = 289.30	*F*(000) = 150
Triclinic, *P*1	*D*_x_ = 1.441 Mg m^−^^3^
Hall symbol: -P 1	Melting point: 401 K
*a* = 3.8453 (13) Å	Mo *K*α radiation, λ = 0.71073 Å
*b* = 8.447 (3) Å	Cell parameters from 230 reflections
*c* = 11.202 (3) Å	θ = 1.9–28.1°
α = 108.672 (6)°	µ = 0.10 mm^−^^1^
β = 97.251 (6)°	*T* = 293 K
γ = 99.772 (6)°	Block, yellow
*V* = 333.29 (19) Å^3^	0.60 × 0.50 × 0.29 mm

### Data collection

**Table:**

Bruker APEXII CCD area-detector diffractometer	1623 independent reflections
Radiation source: fine-focus sealed tube	1198 reflections with *I* > 2σ(*I*)
graphite	*R*_int_ = 0.016
ω–scans	θ_max_ = 28.4°, θ_min_ = 2.0°
Absorption correction: multi-scan *SADABS* (Bruker, 2007)	*h* = −5→4
*T*_min_ = 0.622, *T*_max_ = 1.000	*k* = −11→11
2301 measured reflections	*l* = −12→14

### Refinement

**Table:**

Refinement on *F*^2^	Primary atom site location: structure-invariant direct methods
Least-squares matrix: full	Secondary atom site location: difference Fourier map
*R*[*F*^2^ > 2σ(*F*^2^)] = 0.058	Hydrogen site location: inferred from neighbouring sites
*wR*(*F*^2^) = 0.177	H-atom parameters constrained
*S* = 1.07	*w* = 1/[σ^2^(*F*_o_^2^) + (0.096*P*)^2^ + 0.0878*P*] where *P* = (*F*_o_^2^ + 2*F*_c_^2^)/3
1623 reflections	(Δ/σ)_max_ < 0.001
100 parameters	Δρ_max_ = 0.31 e Å^−^^3^
0 restraints	Δρ_min_ = −0.26 e Å^−^^3^

### Special details

**Table:**

Geometry. All esds (except the esd in the dihedral angle between two l.s. planes) are estimated using the full covariance matrix. The cell esds are taken into account individually in the estimation of esds in distances, angles and torsion angles; correlations between esds in cell parameters are only used when they are defined by crystal symmetry. An approximate (isotropic) treatment of cell esds is used for estimating esds involving l.s. planes.
Refinement. Refinement of *F*^2^ against ALL reflections. The weighted *R*-factor *wR* and goodness of fit *S* are based on *F*^2^, conventional *R*-factors *R* are based on *F*, with *F* set to zero for negative *F*^2^. The threshold expression of *F*^2^ > 2σ(*F*^2^) is used only for calculating *R*-factors(gt) *etc*. and is not relevant to the choice of reflections for refinement. *R*-factors based on *F*^2^ are statistically about twice as large as those based on *F*, and *R*- factors based on ALL data will be even larger.

### Fractional atomic coordinates and isotropic or equivalent isotropic displacement parameters (Å^2^)

**Table:**

	*x*	*y*	*z*	*U*_iso_*/*U*_eq_	Occ. (<1)
O1	1.3180 (5)	1.09147 (19)	0.33644 (14)	0.0620 (6)	
N1	0.7459 (5)	0.6838 (2)	0.16816 (16)	0.0402 (4)	
N2	0.9591 (5)	1.1260 (2)	0.10902 (16)	0.0377 (4)	0.50
C9	0.9591 (5)	1.1260 (2)	0.10902 (16)	0.0377 (4)	0.50
H9A	0.9331	1.2127	0.1798	0.045*	0.50
C1	0.9713 (5)	0.8073 (2)	0.26845 (17)	0.0339 (4)	
C2	1.0530 (6)	0.7939 (3)	0.38842 (19)	0.0435 (5)	
H2A	1.2108	0.8826	0.4556	0.052*	
C3	0.8934 (7)	0.6452 (3)	0.4057 (2)	0.0517 (6)	
H3A	0.9378	0.6331	0.4855	0.062*	
C4	0.6689 (7)	0.5157 (3)	0.3033 (2)	0.0524 (6)	
H4A	0.5633	0.4134	0.3120	0.063*	
C5	0.6028 (6)	0.5402 (3)	0.1870 (2)	0.0485 (6)	
H5A	0.4502	0.4519	0.1181	0.058*	
C6	1.1320 (5)	0.9691 (2)	0.24786 (17)	0.0372 (5)	
C7	1.0544 (5)	0.9809 (2)	0.11607 (17)	0.0333 (4)	
C8	1.0969 (5)	0.8553 (2)	0.00732 (18)	0.0369 (5)	
H8A	1.1642	0.7573	0.0141	0.044*	

### Atomic displacement parameters (Å^2^)

**Table:**

	*U*^11^	*U*^22^	*U*^33^	*U*^12^	*U*^13^	*U*^23^
O1	0.0862 (13)	0.0422 (8)	0.0381 (8)	−0.0157 (8)	−0.0097 (8)	0.0113 (7)
N1	0.0422 (10)	0.0355 (8)	0.0394 (9)	−0.0001 (7)	0.0059 (7)	0.0136 (7)
N2	0.0467 (11)	0.0295 (8)	0.0341 (9)	0.0035 (7)	0.0096 (7)	0.0092 (7)
C9	0.0467 (11)	0.0295 (8)	0.0341 (9)	0.0035 (7)	0.0096 (7)	0.0092 (7)
C1	0.0360 (10)	0.0330 (9)	0.0334 (9)	0.0065 (7)	0.0077 (7)	0.0128 (7)
C2	0.0538 (13)	0.0404 (10)	0.0373 (10)	0.0106 (9)	0.0065 (9)	0.0157 (8)
C3	0.0712 (16)	0.0510 (12)	0.0471 (12)	0.0209 (11)	0.0180 (11)	0.0299 (10)
C4	0.0632 (15)	0.0379 (10)	0.0671 (15)	0.0110 (10)	0.0239 (12)	0.0291 (10)
C5	0.0511 (13)	0.0355 (10)	0.0534 (13)	−0.0015 (9)	0.0097 (10)	0.0140 (9)
C6	0.0421 (11)	0.0328 (9)	0.0326 (9)	0.0012 (8)	0.0041 (8)	0.0108 (7)
C7	0.0333 (10)	0.0298 (8)	0.0335 (9)	−0.0016 (7)	0.0047 (7)	0.0116 (7)
C8	0.0418 (11)	0.0302 (8)	0.0380 (10)	0.0041 (7)	0.0075 (8)	0.0132 (7)

### Geometric parameters (Å, °)

**Table:**

O1—C6	1.216 (2)	C3—C4	1.372 (3)
N1—C5	1.336 (3)	C3—H3A	0.9300
N1—C1	1.341 (2)	C4—C5	1.383 (3)
N2—C8^i^	1.358 (2)	C4—H4A	0.9300
N2—C7	1.360 (3)	C5—H5A	0.9300
N2—H9A	0.9207	C6—C7	1.507 (2)
C1—C2	1.386 (3)	C7—C8	1.385 (3)
C1—C6	1.501 (3)	C8—C9^i^	1.358 (2)
C2—C3	1.385 (3)	C8—N2^i^	1.358 (2)
C2—H2A	0.9300	C8—H8A	0.9300
			
C5—N1—C1	116.84 (17)	C5—C4—H4A	120.6
C8^i^—N2—C7	118.59 (16)	N1—C5—C4	123.62 (19)
C8^i^—N2—H9A	118.5	N1—C5—H5A	118.2
C7—N2—H9A	123.0	C4—C5—H5A	118.2
N1—C1—C2	123.47 (17)	O1—C6—C1	120.89 (17)
N1—C1—C6	117.03 (16)	O1—C6—C7	119.68 (16)
C2—C1—C6	119.48 (17)	C1—C6—C7	119.42 (15)
C3—C2—C1	118.27 (19)	N2—C7—C8	120.92 (17)
C3—C2—H2A	120.9	N2—C7—C6	116.89 (16)
C1—C2—H2A	120.9	C8—C7—C6	122.09 (16)
C4—C3—C2	119.06 (19)	C9^i^—C8—C7	120.49 (17)
C4—C3—H3A	120.5	N2^i^—C8—C7	120.49 (17)
C2—C3—H3A	120.5	C9^i^—C8—H8A	119.8
C3—C4—C5	118.71 (18)	N2^i^—C8—H8A	119.8
C3—C4—H4A	120.6	C7—C8—H8A	119.8
			
C5—N1—C1—C2	−1.5 (3)	C2—C1—C6—C7	177.66 (17)
C5—N1—C1—C6	−179.82 (19)	C8^i^—N2—C7—C8	0.5 (3)
N1—C1—C2—C3	0.0 (3)	C8^i^—N2—C7—C6	177.03 (17)
C6—C1—C2—C3	178.27 (19)	O1—C6—C7—N2	−45.2 (3)
C1—C2—C3—C4	1.6 (3)	C1—C6—C7—N2	133.4 (2)
C2—C3—C4—C5	−1.6 (4)	O1—C6—C7—C8	131.2 (2)
C1—N1—C5—C4	1.5 (3)	C1—C6—C7—C8	−50.2 (3)
C3—C4—C5—N1	0.0 (4)	N2—C7—C8—C9^i^	−0.5 (3)
N1—C1—C6—O1	174.7 (2)	C6—C7—C8—C9^i^	−176.85 (17)
C2—C1—C6—O1	−3.7 (3)	N2—C7—C8—N2^i^	−0.5 (3)
N1—C1—C6—C7	−4.0 (3)	C6—C7—C8—N2^i^	−176.85 (17)

Symmetry codes: (i) −*x*+2, −*y*+2, −*z*.
